# Impaired fertility in adenomyosis: a murine model reveals endometrial receptivity and progesterone resistance imbalances

**DOI:** 10.1530/REP-24-0019

**Published:** 2024-04-17

**Authors:** Marlyne Squatrito, Julie Vervier, Jules Bindels, Laëtitia Bernet, Silvia Blacher, Michelle Nisolle, Carine Munaut

**Affiliations:** 1 Laboratory of Biology of Tumor and Development, GIGA-Cancer, University of Liège, Liège, Belgium; 2 Department of Obstetrics and Gynecology, Hôpital de la Citadelle, University of Liège, Liège, Belgium

## Abstract

**In brief:**

The impact of adenomyosis on reproductive health needs to be fully understood. By using a murine model, this study provides novel insights into the nuanced mechanisms associated with fertility challenges and offers a foundation for targeted interventions.

**Abstract:**

This study investigates the intricate relationship between adenomyosis and reproductive health using a murine model, offering novel insights into this prevalent gynecological disorder. Adenomyosis, characterized by the invasive growth of endometrial tissue into the myometrium, is believed to negatively impact fertility. However, the challenge lies in disentangling this influence, as adenomyosis often coexists with other gynecological diseases. A tamoxifen-induced mice model presents a significant advantage by enabling the specific study of adenomyosis, devoid of confounding influences of concurrent gynecological diseases such as endometriosis. Focusing exclusively on adenomyosis, our study aims to elucidate pathogenic mechanisms underlying fertility issues, focusing on estrous cyclicity, ovarian follicle development, and overall fertility. Our findings uncover disruptions in estrous cyclicity, characterized by an increased duration of time spent in the estrus phase in adenomyosis-induced mice. These disturbances are potentially linked to observed compromised folliculogenesis and the remarkable reduction in litter number and size in mice affected by adenomyosis. Moreover, this study unveils potential drivers of subfertility such as progesterone resistance and altered endometrial receptivity. Within the uteri of mice with adenomyosis, reduced expression of the progesterone receptor and a decreased expression of two implantation-related markers (HoxA10 and integrin β3) were observed. This comprehensive examination sheds light on the nuanced complexities of adenomyosis-associated reproductive challenges, providing a foundation for targeted interventions in addressing fertility issues related to this disease.

## Introduction

Adenomyosis is a benign estrogen-dependent uterine disease characterized by ectopic endometrial glands and stroma within the myometrium and surrounded by smooth-muscle hyperplasia reaction (Bird *et al.* 1972). Two principal forms of the disease, focal and diffuse, are generally described depending on the distribution pattern of the ectopic endometrial implants in the myometrium. Focal adenomyosis is a circumscribed nodular collection whereas diffuse adenomyosis is characterized by different groups of endometriotic glands and stroma distributed throughout the myometrium. This gynecological disorder is often associated with pain (dysmenorrhea, pelvic pain, or dyspareunia), abnormally heavy menstrual bleeding, and poor pregnancy outcomes (infertility, repeated implantation failure, miscarriage, or obstetric complications). However, up to one-third of patients can be asymptomatic (Levgur *et al.* 2000). Accurate prevalence assessment remains challenging due to the lack of standardized diagnostic criteria, protocols, and patient populations (Abbott 2017, Loring *et al.* 2021). Nevertheless, numerous studies suggest a prevalence ranging from 20% to 35% (Abbott 2017). Historically, the gold standard for the diagnosis of adenomyosis was through histological examination of the uterus after hysterectomy. Therefore, it was considered to be a disease affecting mainly multiparous women over 40 years old. Recent advances in imaging techniques, such as transvaginal ultrasound (TVS) and magnetic resonance imaging (MRI), have led to increased diagnoses in young women of reproductive age (Vannuccini & Petraglia 2019) as well as in a population of women undergoing assisted reproductive technologies (Puente *et al.* 2016). One of the most determinant fertility factors is age. In Europe, we observed for the past years a trend to delay the first pregnancy (mean age 29.4 years old) (Fertility statistics – Statistics Explained (europa.eu)). Since adenomyosis could further affect the fertility of those women, understanding the mechanisms underlying adenomyosis development and addressing its impact on fertility outcomes is now crucial (Donnez *et al.* 2021).

Given that adenomyosis often coexists with other gynecological diseases such as endometriosis (20-80%) and uterine fibroids (15-57%), the direct causal relationship between adenomyosis and infertility remains challenging to establish (Sharma *et al.* 2019, Vannuccini & Petraglia 2019, Yu *et al.* 2020, Szubert *et al.* 2021). Furthermore, it is uncertain whether these concurrent conditions act independently or cumulatively to affect fertility (Vercellini *et al.* 2023). For instance, a meta-analysis by Vercellini *et al.*, including 1865 women, indicated that adenomyosis could reduce clinical pregnancy rates and increase miscarriage risk in IVF/ICSI outcomes (Vercellini *et al.* 2014). In addition, among infertile women and, more specifically, those experiencing recurrent miscarriages or repeated implantation failure in Assisted Reproductive Technology (ART), the prevalence of adenomyosis was estimated at 25%. These observations support the possibility that adenomyosis may have a deleterious impact on human reproduction (Puente *et al.* 2016). Various mechanisms have been proposed to explain subfertility and poor reproductive outcomes in adenomyosis, including aberrant uterotubal sperm transport and contractility, altered endometrial function and receptivity, excessive free radical formation affecting oocyte quality and embryo development as well as uterine immune imbalances (Harada *et al.* 2016, Sharma *et al.* 2019). However, the potential confounding effect of endometriosis could not be excluded (Vercellini *et al.* 2014). The use of a murine model of adenomyosis is beneficial for studying fertility outcomes associated with adenomyosis without associated pathologies such as myomas or endometriosis. Among the various models available to induce adenomyosis, neonatal treatment of CD1 mice with tamoxifen, a selective estrogen receptor modulator, is a model of choice because it appears to reliably produce severe adenomyosis lesions in a high proportion of mice and has been used extensively for examining adenomyosis pathogenesis and drug screening (Green *et al.* 2005, Yen *et al.* 2017*a*, Marquardt *et al.* 2020).

In this study, we aimed to elucidate the potential pathogenic mechanisms through which adenomyosis, in the absence of associated gynecological disorders, could impact pregnancy outcomes negatively. We assessed estrous cycles, folliculogenesis, and the expression of genes related to endometrial receptivity as well as progesterone resistance in a murine model. This would allow us to explore the multifactorial factors contributing to infertility associated with adenomyosis.

## Materials and methods

### Mouse model of adenomyosis

Animal experiments in this study were approved by the Animal Ethics Committee of the University of Liège (#2387) and conducted following relevant guidelines and regulations. In total, 69 female neonatal CD1 mice (born from pregnant mice provided by Charles River Laboratories (Italy)) were orally dosed with 2,7 µmol/kg tamoxifen (= 1 mg/kg) (Sigma, T5648) suspended in a peanut oil/lecithin/condensed milk mixture (2:0.2:3, v/v) on days 2 through 5 after birth (day of birth = day 1) at a dose volume of 5 µL/g body weight (Green *et al.* 2005). Seventy-eight female neonatal control mice received vehicle only. All mice were housed under controlled temperature (+/- 21°C) and light (12-h light–12-h darkness cycle) with ad libitum access to food and water. Animals were euthanized either at 1-month-old (CTL, *n* = 12; ADM, *n* = 14) or 3-month-old (CTL, *n* = 66; ADM, *n* = 55). Ovaries were collected and weighed; one was fixed in 4% formalin while the second was snap-frozen in liquid nitrogen. Uteri from 1 and 3-month-old mice were also removed and weighted. One of the uterine horns was divided into 2 (1-month-old mice) or 3 parts (3-month-old mice) and snap-frozen for RNA and protein extraction. The second horn was fixed in 4% formalin. Complete autopsies were performed. Histopathological examinations of ovaries and uteri were conducted using standard histological sections prepared from formalin-fixed material and stained with hematoxylin and eosin.

To study progesterone resistance and implantation gene expression, only mice in the proliferative phase of the estrus cycle (proestrus or estrus phase) were included. To synchronize the phase of the cycle of the mice included in this aim, we injected them subcutaneously for 3 consecutive days with 17β-estradiol (100 ng/100 µL) before sacrifice.

### Vaginal smears analysis

The estrous cycle phase was determined once daily (between 08:00 and 10:00 h) for 14 consecutive days by collecting samples via vaginal lavage as previously described by Caligioni *et al.* (Caligioni 2010). Briefly, the vagina was gently flushed with 30 µL sterile PBS using a sterile 200 µL pipette tip. The collected PBS was deposited on a glass slide as a drop, dried, and fixed for 30 min in 100% ethanol. Ethanol-fixed smears were then rehydrated and stained with hematoxylin–eosin. The estrous cycle stage was determined by examining the proportion and morphology of leukocytes and epithelial cells under a 40× objective light microscope. When the female is in proestrus, mostly nucleated epithelial cells are present. More cornified epithelial cells are present as the cycle stage advances to estrus. If the female is not pregnant, metestrus will begin. Metestrus is a brief stage during which the uterine lining will begin to slough, and leads to the presence of cornified epithelial cells and leukocytes present in vaginal smears. Diestrus is the longest stage characterized by leukocytes in a very high density. If a smear appears to contain more than one cell type, the stage representing the majority cell type is selected (Byers *et al.* 2012). In total, 18 mice in the adenomyosis group and 25 in the control group were subjected to vaginal lavage for our first experiment. We repeated the experiment with vaginal smears on 19 other adenomyosis-induced mice and 25 control mice to validate our results.

### Fertility and litter size analysis

To analyze the fertility, 12 female mice (3-month-old) tamoxifen-treated (*n* = 6) or vehicle-treated (*n* = 6) during the neonatal period were bred with CD1 fertility-proven male mice (1:1). Mice were mated continuously for 3 months, and litter number and size were monitored. To calculate the average litter size at weaning, the total number of pups born during the breeding trial was divided by the total number of litters. To ensure that a lack of conception was not due to male subfertility, male mice breaded with control mice were exchanged with male mice mated with tamoxifen-treated mice after a first successful mating cycle.

### Immunohistochemistry

Ovarian sections were assessed by immunostaining serial sections with antibodies against Lhx8 to facilitate the quantification of follicles. Uterine sections of 1- or 3-month-old mice were stained in visible light with three markers of implantation (HoxA10, integrin β3, and LIF). Progesterone resistance was also assessed by double immunofluorescence staining for αSMA and PGR. Briefly, ovarian and uterine sections were deparaffinized and rehydrated, followed by antigen retrieval using an autoclave (11 min, 126°C, 1.3 bar). After cooling down for 20 min, endogenous peroxidase activity was blocked by incubating the sections in 3% hydrogen peroxide for 20 min at room temperature (RT). Non-specific binding sites were blocked by incubation in the ‘Animal-Free Blocking Solution’ (Cell Signaling) for 20 min at RT. Primary antibodies (Table 1) were diluted at different concentrations in the ‘REAL antibody diluent’ (Dako) and incubated for 1 h at RT except for cleaved HoxA10, integrin β3, and LIF which were incubated overnight at 4°C. For double immunostaining, the anti-PGR and anti-αSMA antibodies were mixed as well as for anti-EpCAM and anti-αSMA antibodies. Afterward, sections were incubated with the secondary antibody linked to horseradish peroxidase (ENVISION/HRP ready to use, Dako) for 30 min at RT. For staining in visible, the revelation was revealed using DAB + (Dako) followed by hematoxylin counterstaining, and sections were mounted using Entellan new mounting medium (Sigma-Aldrich). For fluorescent staining, the fluorescein tyramide kit (PerkinElmer) was used for 10 min and sections were mounted with DAPI Fluoromount-G mounting medium (SouthernBiotech, Birmingham, AL, USA). Stained sections were then scanned using the NanoZoomer2.0HT digital slide scanner (Hamamatsu, Photonics, K.K., Japan) or the SLIDEVIEW VS200 research slide scanner (Olympus, Anvers, Belgium) equipped with a UPlan-XApo 20× 0.8 NA objective (Olympus).

**Table 1:** Antibodies for immunostaining.

Primary antibody	Conjugate	Catalogue number	Dilution^†^	Secondary antibody*
Lhx8	–	Abcam, ab51519	1:100	ENVISION/HRP
EpCAM	–	Cell Signaling, 93790	1:400	ENVISION/HRP
PGR A/B	–	Cell Signaling, 3153	1:100	ENVISION/HRP
HoxA10	–	Cell ignaling, 58891	1:100	ENVISION/HRP
Integrin β3	–	Affinity, AF6086	1:200	ENVISION/HRP
LIF	–	Affinity, DF13730	1:50	ENVISION/HRP
αSMA	FITC	Sigma, F3777	1:400	–

^†^Dilution of primary antibody; *Anti-rabbit secondary antibody ENVISION/HRP ready to use.

### Quantification of PGR immunostaining

**Figure 1:**
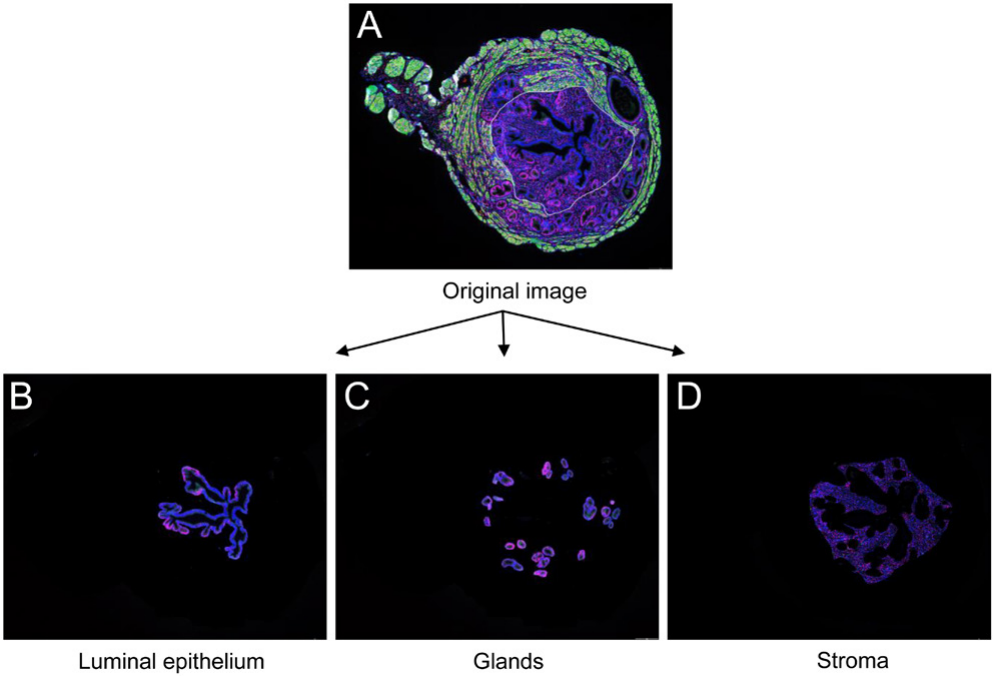
Image processing workflow to quantify endometrial PGR expression in uterus. Original image (A) from the whole scanned image registered in RGB space decomposes in uterine surface epithelium (B), endometrial glands (C), and stroma (D).

Image processing and automatic quantification of PGR expression in the endometrium were performed using the image analysis toolbox of MATLAB R2021b according to the following steps: the original images were registered in the full-color red, green, and blue (RGB) space where PGR labeling appeared in red, αSMA in green and DAPI nuclear staining in blue (Fig. 1A). Three regions of interest were drawn manually: (a) the total studied region (b) the external border of the luminal epithelium, and (c) the glands. This allows to decompose the original image into three ones corresponding to the luminal epithelium (Fig. 1B), the glands (Fig. 1C), and the stroma (Fig. 1D). For each image, blue and red components were binarized independently using an automatic threshold (Otsu *et al.* 1979), and the area occupied by the blue and red cells were calculated. Finally, the ratio between red and blue cells was determined (area occupied by red cell/area occupied by blue cells).

### Follicle quantification

Fixed ovarian samples were embedded in paraffin and then sectioned into 5 µm thickness pieces. One in every ten sections was stained for LIM-homeobox protein 8 (Lhx8) transcription factor for differential follicle counts. Lhx8 is an oocyte-specific transcription factor that is essential in regulating postnatal folliculogenesis, especially in the earliest stages of primordial follicle activation. It can be used as a biomarker for rapid assessment of follicle pool in the mouse ovary. Scanned sections labeled for Lhx8 were analyzed using the NDP.view2 software (Hamamatsu Photonics K.K., Hamamatsu, Japan). Follicles were classified into primordial, primary, and secondary or more growing according to morphological mouse follicle classification described by Myers *et al.* (Myers *et al.* 2004). Total follicle density was defined as the number of follicles per mm^2^ (n/mm^2^) after manually calculating the ovarian surface. Results are expressed as the number of each follicle type per mm^2^. At least six sections per ovary were quantified blindly. The number of corpora lutea was determined across sections encompassing the eight sections of the ovary to avoid double counting. Raw counts of corpora lutea were not adjusted.

### Western blot analysis

Follicular development was assessed by Western blot analysis of folliculogenesis factors. One ovary per mouse was used for protein extraction. The protein extraction was performed with radioimmunoprecipitation assay (RIPA) buffer containing 4% of a protease and phosphatase inhibitor (Roche). Lysate was collected, and protein concentrations were determined using a protein assay kit (Bio-Rad Laboratories). Equal amounts of protein (20 µg) were denatured and separated by electrophoresis on SDS–polyacrylamide gels. According to standard procedures, proteins were transferred onto a polyvinylidene difluoride membrane (PerkinElmer). The membrane was then blocked using 5% non-fat milk solution during 2 h on a shaker, then incubated with respective primary antibodies (Cell Signaling), at a dilution of 1/1000, in the blocking solution, overnight at 4°C. Primary antibodies used were anti-FOXL2 (Novus, NB100-1277), anti-BMP15 (Biorbyt, Orb247897), and anti-FSHR (Proteintech, 22665-1-AP). The appropriate horseradish peroxidase-conjugated secondary antibody was added to the membrane, followed by a 1h incubation at RT. After sequential washing of the membranes to remove excess secondary antibodies, signals were detected using an enhanced chemiluminescence (ECL) kit (PerkinElmer) according to the manufacturer’s instructions in a LAS4000 imager (Fujifilm, Tokyo, Japan). The intensities of the protein bands were quantified using QuantityOne Analysis software. Data are expressed as the fold-change compared to the control group. HSP70 expression was measured to verify equal loading.

### Real-time PCR analysis of mRNA

The expression of *Pgr*, *HoxA10*, integrin β3 (*Itgb3*), and *Lif* mRNA in uterine tissues (one-third of one horn for 3-month-old mice and half horn for 1-month-old mice) was determined by real-time PCR. According to the manufacturer’s instructions, RNA from individual mice was isolated using an RNeasy® mini kit (Qiagen). For each sample, 1 µg of RNA was converted to cDNA using the FastGene Scriptase II Ready Mix (Nippon Genetics, Düren, Germany). The quantitative RT-PCR was performed using SYBR Green PCR Master Mix and specific primers (Table 2) in a Fast Real-time PCR (QuantStudio 3, Thermo Fisher Scientific). Relative gene expression was analyzed according to the 2^−ΔΔCt^ method. All samples were assayed in duplicate reactions. The *Rplp0* and *Gapdh* genes were used as housekeeping genes to normalize the expression level, as Lin *et al.* advised for mouse uterus (Lin *et al.* 2013).

**Table 2:** Primers for RT-qPCR.

Gene name	GenBank number	Primer sequences (5′–3′)		Product size (bp)
		Forward	Reverse	
*Pgr*	NM_008829.2	CTACTCGCTGTGCCTTACCATG	CTGGCTTTGACTCCTCAGTCCT	139
*Hoxa10*	NM_008263.4	GGCAGTTCCAAAGGCGAAAAT	GTCTGGTGCTTCGTGTAAGGC	86
*Itgb3*	NM_016780.2	GGCGTTGTTGTTGGAGAGTC	CTTCAGGTTACATCGGGGTCA	138
*Lif*	NM_008501.3	GCTGTATCGGATGGTCGCATA	CACAGACGGCAAAGCACATT	156
*Gapdh*	NM_001289726.2	GGTGGACCTCATGGCCTACA	CTCTCTTGATCAGTGTCCTTGCT	82
*Rplp0*	NM_007475.5	GGACCCGAGAAGACCTCCTT	GCACATCACTCAGAATTTCAATGG	85

### Statistical analysis

Statistical analyses were performed using GraphPad Prism software (GraphPad), using a Mann–Whitney test to compare two groups or a two-way ANOVA to calculate the cumulative number of pups over time. All data are presented as mean ± s.e.m . or median with interquartile range. A probability of *P* ≤ 0.05 was considered to be statistically significant.

## Results

### Most female mice develop severe adenomyosis 3 months after neonatal tamoxifen treatment

**Figure 2:**
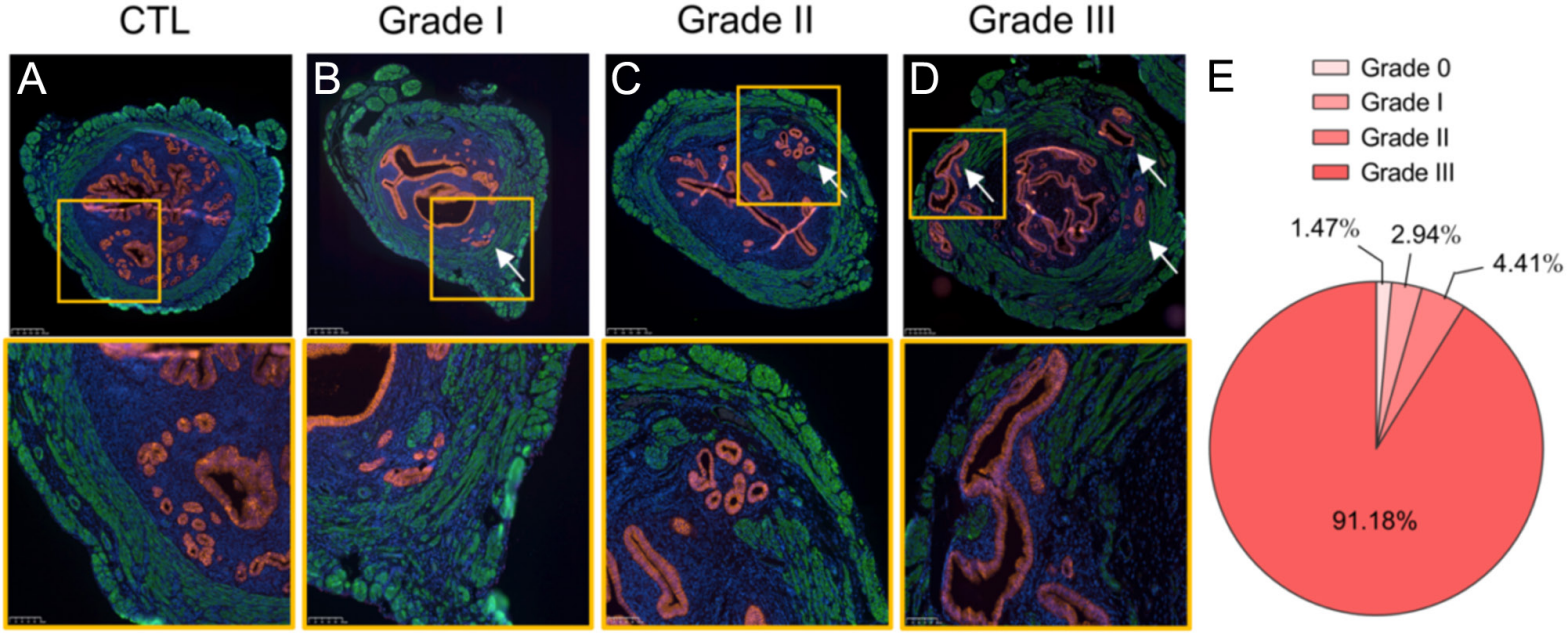
Classification of adenomyosis progression in an experimentally induced mouse model. Representative microscopic images of the uterus from (A) untreated mice, (B) grade I, (C) grade II, and (D) grade III adenomyosis. Red staining = EpCAM; green staining = aSMA; blue staining = DAPI. The arrow marks the ectopic endometrium. (E) Pie chart representing the proportion of mice who have developed adenomyosis with grade 0, I, II, or III.

The grade of adenomyosis was evaluated using criteria previously described by Bird *et al.* (Bird *et al.* 1972). This classification is based on the depth of myometrial infiltration of adenomyotic foci on histological sections by grading the severity according to adenomyotic involvement of the inner third (grade I, superficial adenomyosis), two-thirds (grade II), or entire myometrium (grade III, deep adenomyosis). In our mice model, we observed a disruption and a distortion of the myometrium of all 3-month-old adenomyosis-induced mice. The invasion of the muscle layer by epithelial and/or stromal endometrial cells was observed in 98.53% of neonatally treated females. Contrary to what is observed in mice with adenomyosis, the myometrium of control mice is composed of two well-defined layers without discontinuity (Fig. 2A). Immunofluorescence analysis of EpCAM co-staining with αSMA allowed us to determine the percentage of each 3-month-old mouse by stage of disease. Only 1.47% of treated mice did not develop adenomyosis. 2.94% and 4.41% have grade I and II adenomyosis, respectively (Fig. 2B, C, and E). Most of the mice (91.18%) are affected by grade III adenomyosis (Fig. 2D and E), which confirmed the successful establishment of the mouse adenomyosis model. The grade of adenomyosis was not assessed using these criteria in 1-month-old mice because of the thin myometrium at this age. In this case, we only considered the presence or absence of myometrial invasion. All 1-month-old tamoxifen-treated mice have developed myometrial disruption (data not shown).

### Adenomyosis-induced mice present disrupted estrous cyclicity

**Figure 3:**
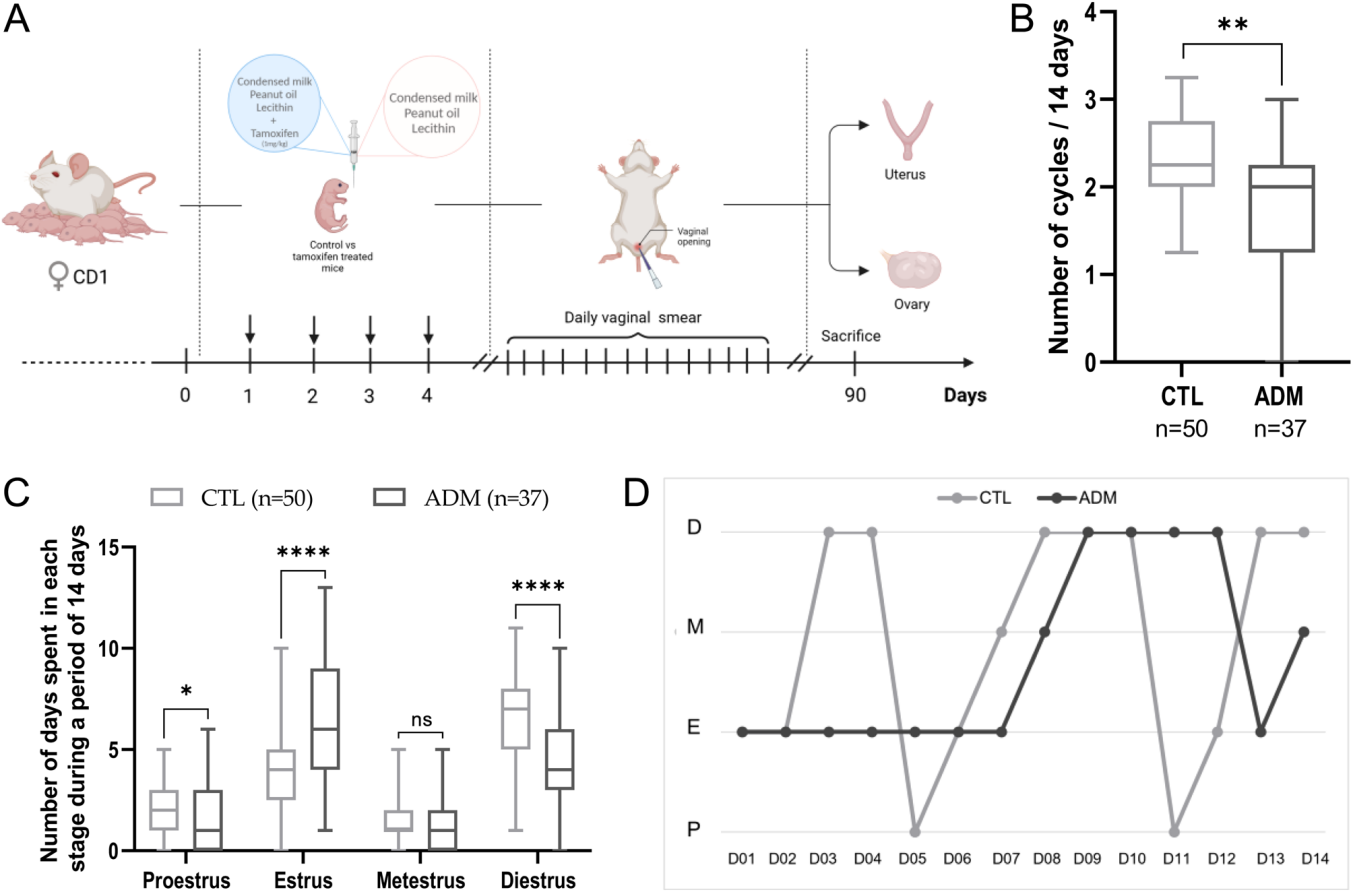
Impact of adenomyosis on estrous cycles. (A) Schematic diagram of the experiment performed on adenomyosis-induced mice model to assess estrous cyclicity. (B) Graph representing the number of cycles during 14 days in tamoxifen-treated mice compared to control mice. (C) Effects of tamoxifen treatment on total number of days spent in each stage during the same period. (D) Representative estrous cycles of control and treated mice as measured by vaginal cytology at 2 months of age (P, pro-estrus; E, estrus; M, metestrus; D, diestrus). The values are the median with the interquartile range. Statistical significance (Mann–Whitney test): *P ≤ 0.05; **P ≤ 0.01; ****P ≤ 0.0001; ns, non-significant.

To study the potential effects of adenomyosis on estrous cyclicity, daily vaginal smears were performed two months after birth in each group of mice (the adenomyosis-induced group and the control group). The smears were collected for 14 consecutive days to assess estrous cycles (Fig. 3A). Adenomyosis-induced females underwent less regular cycles than control mice (*P* = 0.0012) (Fig. 3B). Furthermore, a significant increase in the percentage of days spent in estrus (*P* ≤ 0.0001) and a concomitant decrease in the rate of days spent in proestrus (*P* = 0.0466) and diestrus (*P* ≤ 0.0001) phase was observed in adenomyosis-induced mice (Fig. 3C). As represented in Fig. 3D, mice with adenomyosis exhibited a prolonged estrous cycle compared to control mice, mainly due to the increased duration of the estrus phase. These data suggested that adenomyosis negatively impacts estrous cyclicity.

### Adenomyosis-induced mice show impaired ovarian follicle development

**Figure 4:**
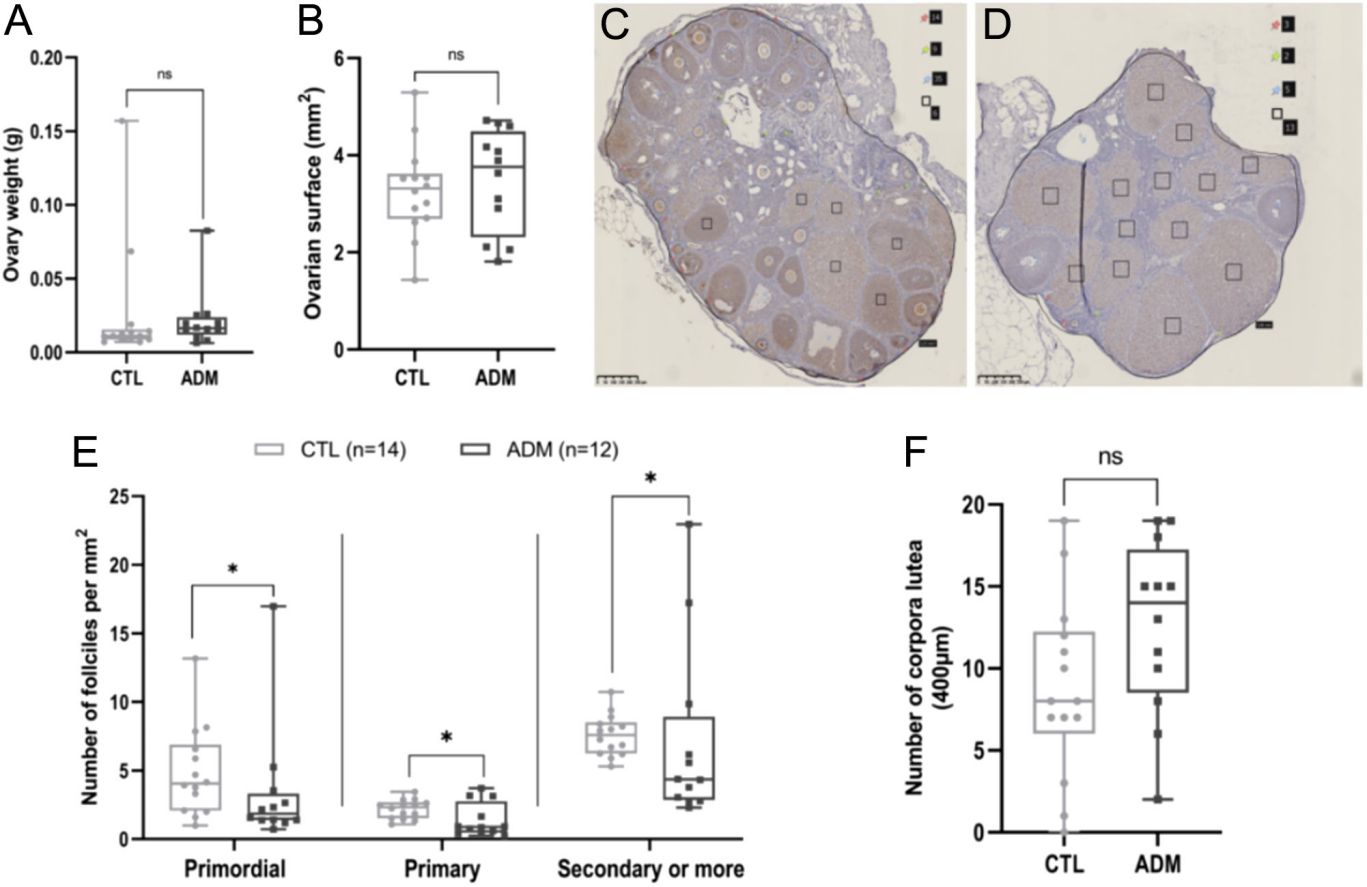
Comparison of ovarian weight, surface, and follicle density between control mice and adenomyosis-induced mice. Comparison of ovary weight (A) and surface (B) between control and mice with adenomyosis. Representative images of Lhx8 staining of (C) control and (D) adenomyosis-induced mice ovaries. Red pins indicate primordial follicles, green pins primary follicles, blue pins secondary or more growing follicles and black rectangle corpora lutea. Follicle density was expressed as the total number of each follicle type per mm^2^ (E). The number of corpora lutea was evaluated on ovarian section of 400 μm (F). The values are the median with the interquartile range. Statistical significance; ns, non-significant; *P ≤ 0.05.

**Figure 5:**
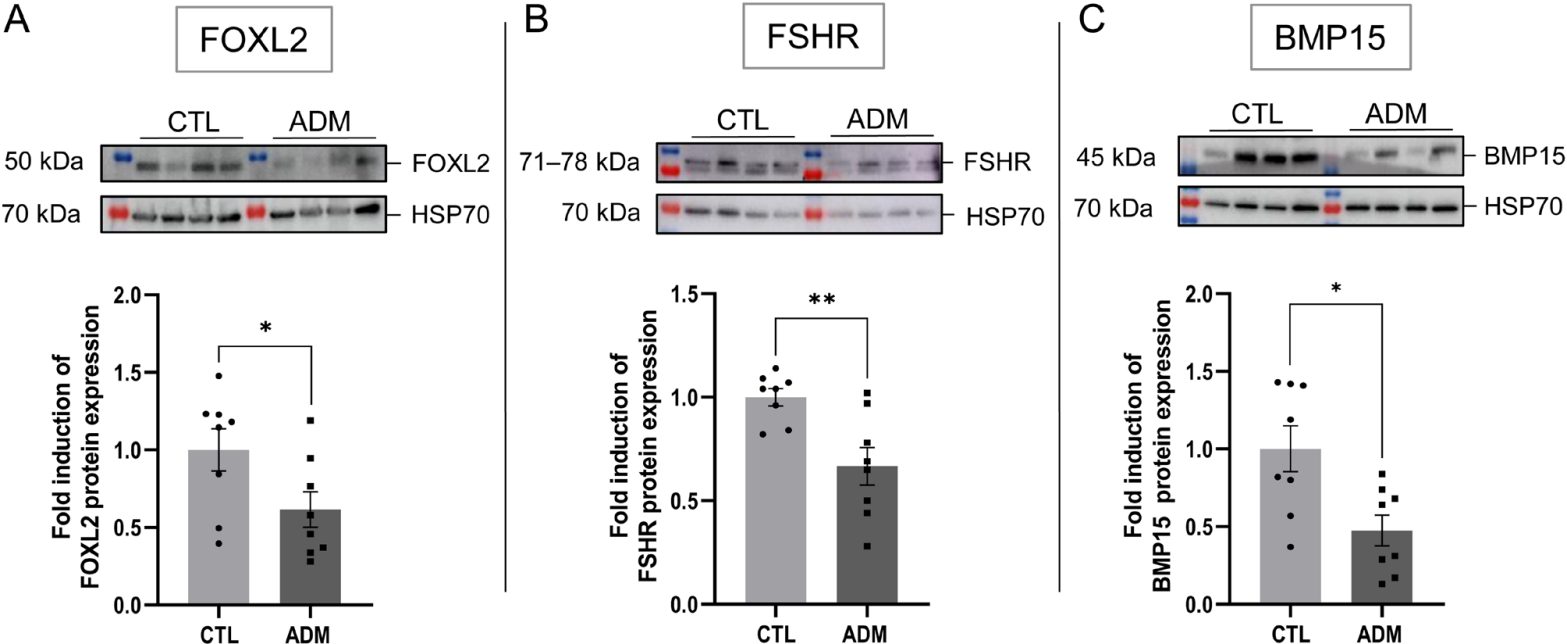
Comparison of ovarian follicle development between control and adenomyosis-induced mice. Representative immunoblots and quantification of FOXL2 (A), FSHR (B), and BMP15 (C) expression in uterus of control and adenomyosisinduced mice. Control group: *n* = 8; adenomyosis group: *n* = 8. The data are mean ± s.e.m .; *P ≤ 0.05; **P ≤ 0.01.

To determine if the disruption in estrous cyclicity was related to altered follicular development, we first analyzed the ovaries of 3-month-old mice. There were no apparent differences in ovarian weight (*P* = 0.1411) and surface area on histological sections (*P* = 0.4397) between adenomyosis-induced and control mice (Fig. 4A and B). Nevertheless, follicular quantification analyses after Lhx8 immunodetection on ovarian sections reveal a reduction in the total number of primordial (*P* = 0.0356), primary (*P* = 0.0393) and secondary or more growing follicles (*P* = 0.0407) per mm^2^ in adenomyosis-induced ovaries (Fig. 4D and E) compared to control ones (Fig. 4C and E). No difference in the number of corpora lutea (*P* = 0.0862) was observed (Fig. 4F). To further investigate how folliculogenesis was impaired in mice affected by adenomyosis, we performed western blot analysis to evaluate the expression of three crucial oocyte-derived factors involved in follicular development: BMP15, FOXL2, and FSHR. The protein levels of all these factors were lower in adenomyosis mice compared with control (FOXL2, *P* = 0.0499; FSHR, *P* = 0.0044; BMP15, *P* = 0.0207; Fig. 5A, B, and C). Both histological and protein analysis suggest that adenomyosis may impair follicle development and reduce ovarian reserve despite preserved ovulation. Adenomyosis-related infertility could therefore be linked to reduced oocyte quality or embryo implantation defects.

### Adenomyosis causes female subfertility

**Figure 6:**
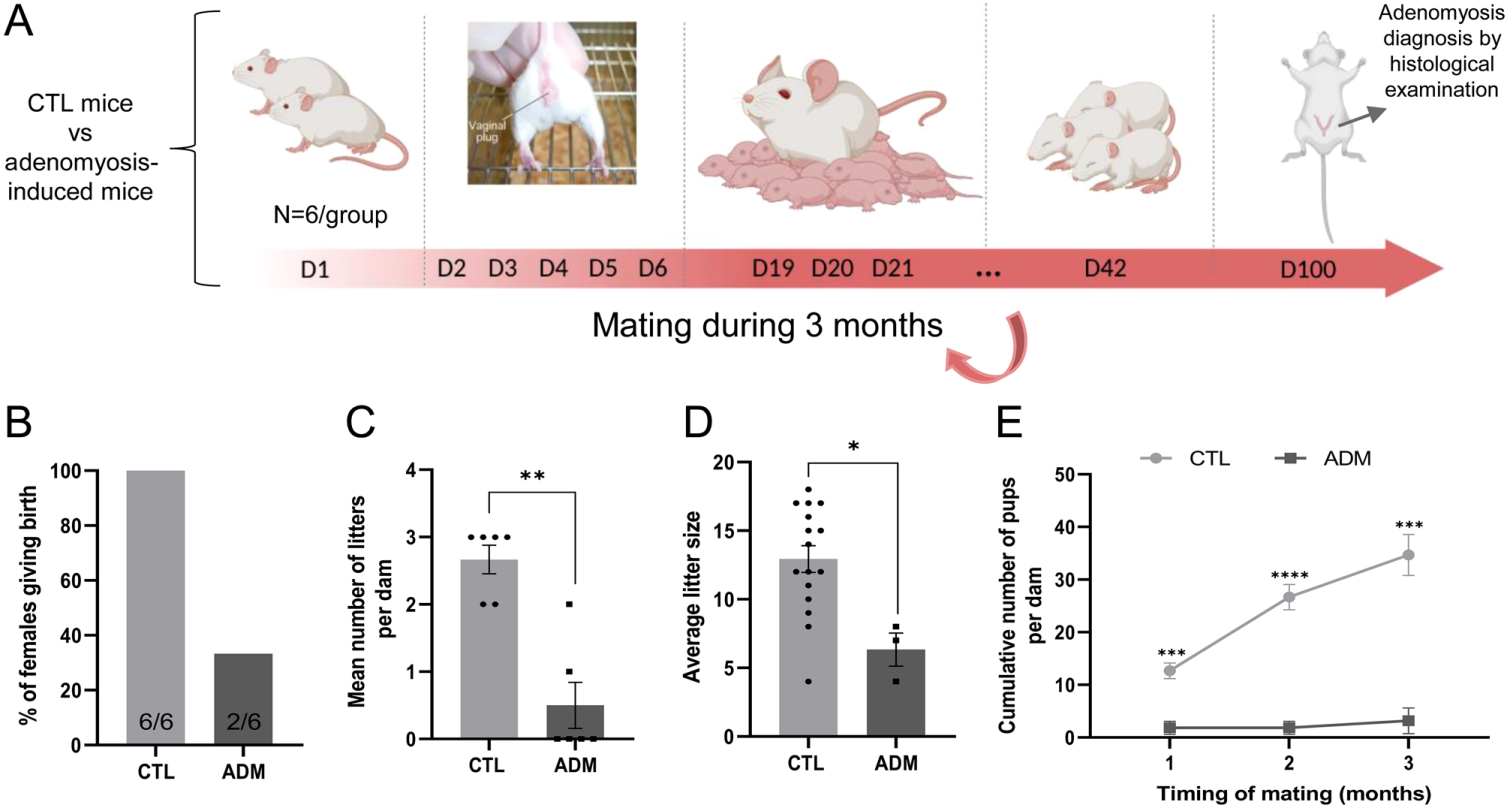
Impact of adenomyosis on fertility outcomes. Schematic diagram of the mating experiment to evaluate how fertility is affected in adenomyosis-induced mice (A). Reduction of the number of pregnant females (B) as well as the litter number (C), litter size (D), and cumulative number of pups (E) of adenomyosis-induced mice. The values are the mean ± s.e.m. Statistical significance (Mann–Whitney test or two-way ANOVA): *P ≤ 0.05; **P ≤ 0.01; ***P ≤ 0.001; ****P ≤ 0.0001.

To further investigate the effect of adenomyosis on fertility, 3-month-old CD1 control and adenomyosis-induced mice were pair-bred with sexually mature (8-week-old) males continuously for 3 months. The number of females giving birth, the mean number of litters and average litter sizes were recorded (Fig. 6A). During this period, 100% of control females have given birth at least once, while only 33.33% of mice in the pathology-induced group delivered (Fig. 6B). In addition, compared with controls, females affected by adenomyosis had reduced litter numbers (*P* = 0.0065) and sizes (Figs 6C and D). Indeed, control mice gave birth to an average of 12.94 ± 0.98 pups, while the average litter size of adenomyosis-induced mice was 6.33 ± 1.20 (*P* = 0.0155) (Fig. 6D). These numbers include only pups that are still alive at weaning. The cumulative number of pups per dam indicates a lower number in the adenomyosis-induced group than in the control group, starting after month 1 of mating (*P* = 0.0007) and persisting until the end of the experiment (*P* = 0.0003) (Fig. 6E).

### Progesterone resistance is a potential mechanism leading to adenomyosis-related infertility

**Figure 7:**
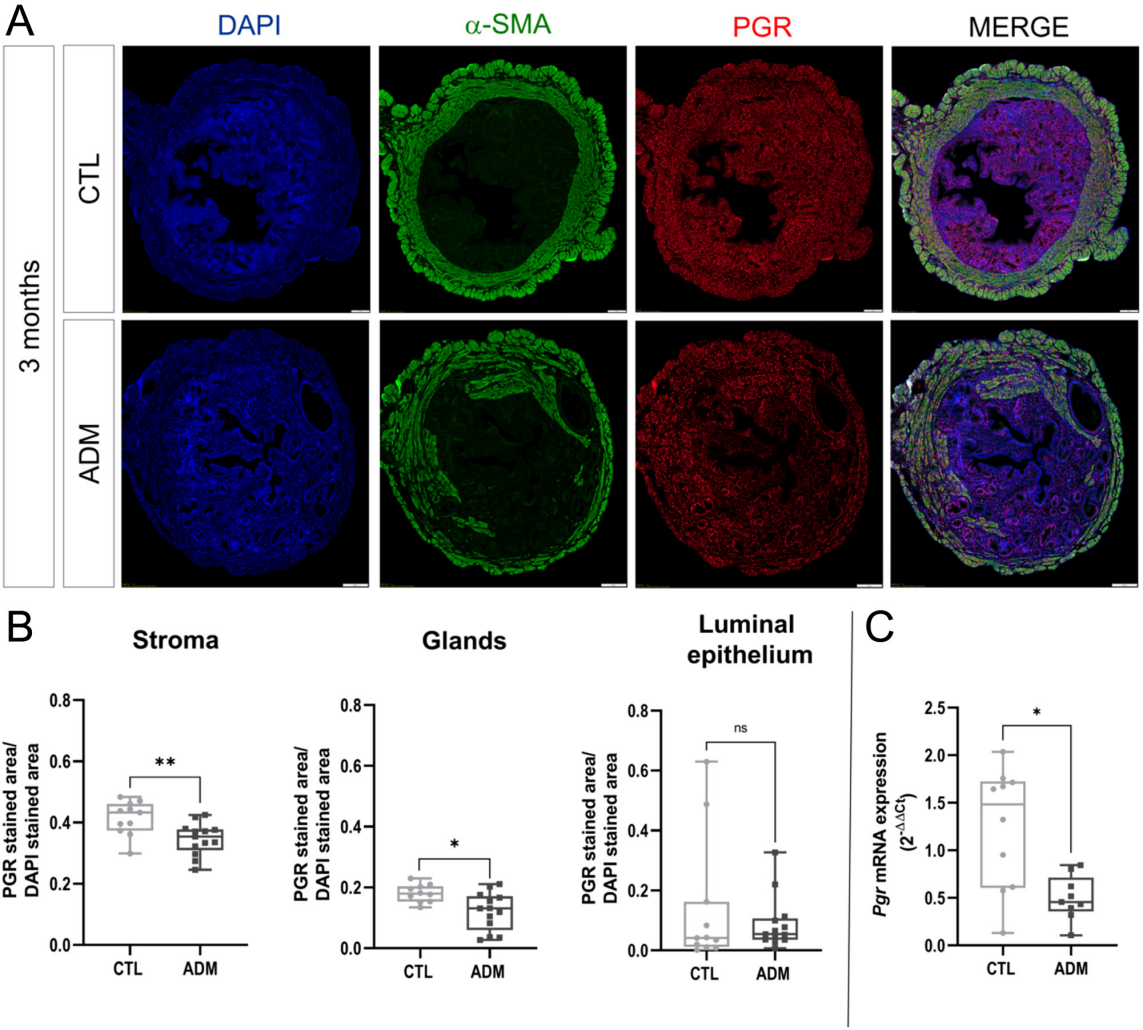
Progesterone receptor (PGR) expression in the uteri of control and adenomyosis-induced mice. Immunofluorescence (A) and computer-assisted quantification (B) of PGR expression in the uteri of 3-month-old control and adenomyosis-induced mice. Bar scale = 200 μm. The mRNA expression levels of Pgr were also assessed (C). The values are the median with the interquartile range. Statistical significance (Mann–Whitney test): *P ≤ 0.05; **P ≤ 0.01; ns, non-significant.

To assess progesterone responsiveness in the uterus of adenomyosis-induced mice, we examined progesterone receptor expression in the eutopic endometrium of the two groups of mice by immunofluorescence. We observed that PGR was expressed in the stromal and epithelial luminal and glandular cell nuclei (Fig. 7A). The results indicate a reduced proportion of PGR-positive stromal (*P* = 0.0048) and glandular (*P* = 0.0178) cells in eutopic endometrium of 3-month-old adenomyosis-induced mice than in control mice. Nevertheless, no difference in PGR expression in luminal epithelial cells was observed (Fig. 7B). The *Pgr* gene expression analysis of entire uteri from 3-month-old mice confirmed the results obtained by immunofluorescence. The progesterone receptor was lower expressed in the uteri of adenomyosis-induced mice compared to control mice (*P* = 0.0101) (Fig. 7C). It can be inferred that subfertility in mice with adenomyosis may be caused by progesterone resistance.

### Adenomyosis impairs endometrial receptivity and implantation

**Figure 8:**
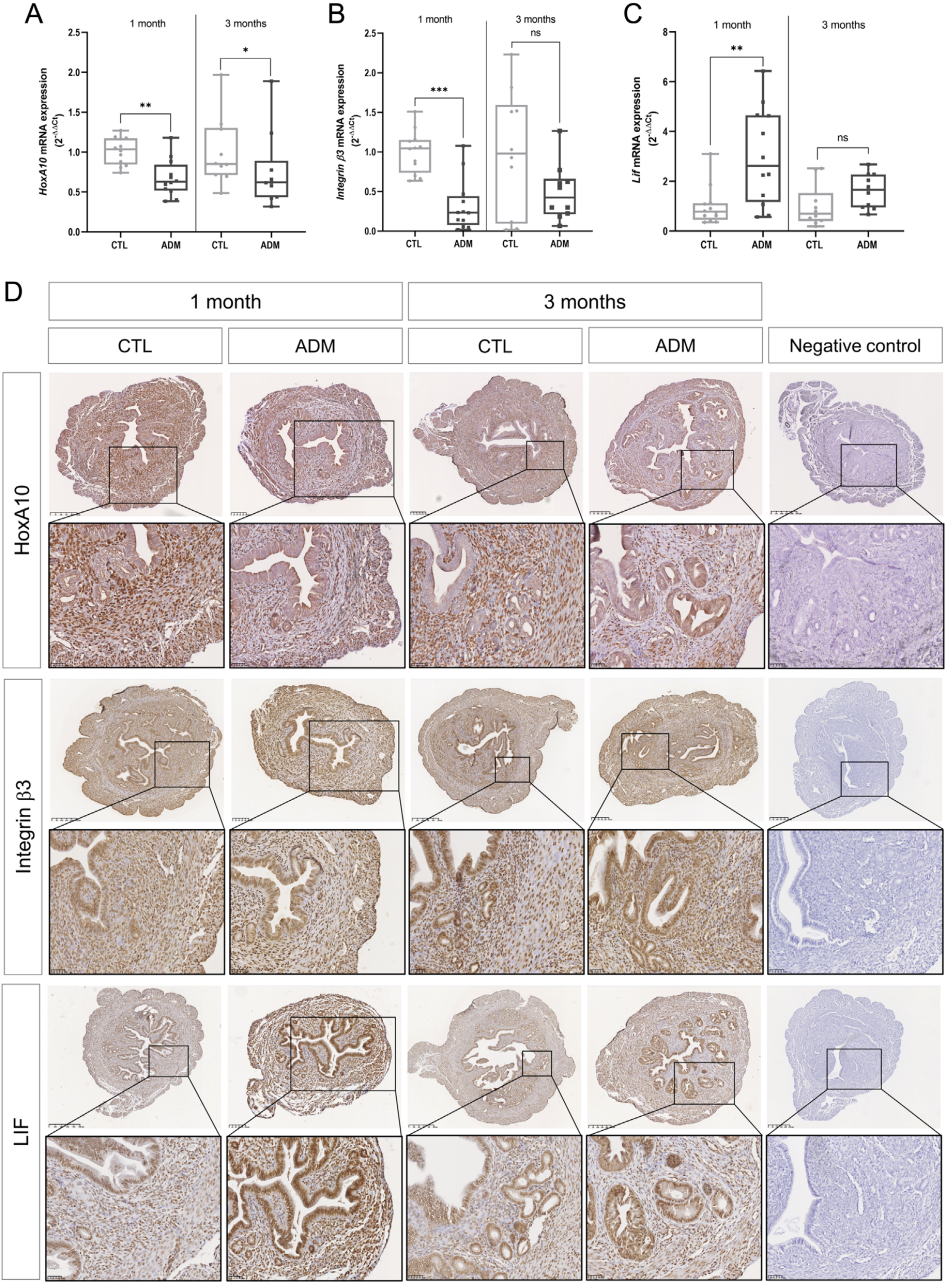
Implantation-related markers expression in the uteri of healthy and adenomyosis-induced mice. The mRNA expression levels of *HoxA10* (A), *Itgb3* (B), and *Lif* (C) in the uteri of 1-month-old and 3-month-old control and adenomyosis-induced mice. Representative immunohistochemistry of *HoxA10*, , and *Lif* uterine expression (D). The values are the median with the interquartile range. Statistical significance (Mann–Whitney test): *P ≤ 0.05; **P ≤ 0.01; ***P ≤ 0.001; ns, non-significant.

The expression of implantation-related markers was measured to explore the mechanisms leading to impaired endometrial receptivity and implantation caused by adenomyosis. The results indicated a reduction of endometrial receptivity-related genes in the 1-month-old mice of the adenomyosis group. In this group, we observed a significant decrease in the expression of *HoxA10* (*P* = 0.0011) and integrin β3 (*P* = 0.0002) mRNA levels in comparison to those in the control group. Those reduced levels of expression are maintained over time for *HoxA10* (*P* = 0.0433) but not for integrin β3 (*P* = 0.2110) (Fig. 8A, B, and C). The IHC staining of these implantation-related markers confirmed the decreased protein expression in the adenomyosis group but also revealed differences in localization. Indeed, *HoxA10* is mainly located in endometrial luminal epithelial cells and glandular epithelial cells. On the contrary, control mice do not express *HoxA10* in epithelial endometrial cells but rather in stromal cells (Fig. 8D). IHC staining of integrin β3 revealed no significant difference between the healthy and adenomyosis mice groups (Fig. 8D). Surprisingly, the uterine mRNA expression of *Lif* was increased in 1-month-old adenomyosis-induced mice (1 month: *P* = 0.0056; 3 months: *P* = 0.0630) (Fig. 8C). These results were confirmed by IHC analysis, which showed an apparent increase in *Lif* expression levels in the glandular and luminal epithelium of the endometrium of mice with adenomyosis compared with control mice (Fig. 8D). Taken altogether, these results suggested that adenomyosis may alter endometrial receptivity through downregulation of implantation-related genes.

## Discussion

The results of this study shed light on the complex relationship between adenomyosis and female reproductive health, encompassing disease severity, estrous cyclicity, ovarian follicle development, fertility, progesterone responsiveness, and endometrial receptivity. These findings contribute to a better understanding of the potential mechanisms underlying adenomyosis-related infertility and the multifactorial impact of this condition.

The analysis of the severity of the adenomyosis induced in our mouse model revealed a high incidence of deep adenomyosis, with over 90% of the treated mice exhibiting grade III adenomyosis. Unlike the well-defined myometrial layers in the healthy uterus, the affected mice displayed a disruption and distortion of the myometrium due to the invasion of epithelial and stromal endometrial cells. Furthermore, all 3-month-old mice developed diffuse adenomyosis. According to Park *et al.*, symptoms experienced by women with diffuse adenomyosis are more pronounced than those with focal adenomyosis (Park *et al.* 2016). This extensive myometrial disruption is also described in human uteri affected by adenomyosis. Those results ensure the successful establishment of the model and its value in simulating the human condition without any associated comorbidities (Upson & Missmer 2020, Loring *et al.* 2021).

In women with adenomyosis, abnormal levels of free radicals have been found in the uterine cavity, which can also negatively impact oocyte quality and inhibit embryo development and implantation, resulting in reduced pregnancy rates (Barroso *et al.* 1998, Harada *et al.* 2016, Vercellini *et al.* 2014). While endometriosis and adenomyosis share similarities, research on the involvement of these mechanisms in adenomyosis is limited (Maruyama *et al.* 2020). Recent studies have primarily focused on endometrial receptivity and function (Hiraoka *et al.* 2023), with little investigation into folliculogenesis and menstrual cycles. In our model, adenomyosis-induced mice displayed disrupted estrous cyclicity, characterized by irregular cycles and prolonged periods in the estrus phase. Furthermore, our analysis of ovarian follicular yield revealed a significant reduction in primordial, primary, and secondary or more growing follicles per unit area in adenomyosis-induced mice compared to controls. In addition to the impact of adenomyosis on folliculogenesis and ovarian reserve, ovarian function may also be impaired. The reduced expression of critical oocyte-derived factors, including Foxl2, BMP15, and FSHR, further indicates ovarian dysfunction in adenomyosis. Several studies have shown that female mice with a deletion of these genes experience subfertility (Desai *et al.* 2013, Abir *et al.* 2014, Zhou *et al.* 2022). In our study, reduced expression of these genes in the ovaries of mice with adenomyosis could partially explain the observed fertility outcomes in the mice model, with only 33.33% of the experimental group giving birth compared to 100% in the control group. The mice with adenomyosis had smaller litter sizes and reduced number of litters. The cumulative number of pups born from adenomyosis-affected mice was, indeed, consistently lower throughout the mating period. These findings align with clinical observations of decreased fertility in women with adenomyosis highlighting the profound impact of the condition on reproductive outcomes (Liang *et al.* 2022).

An essential role of the ovaries is to produce sex hormones in response to stimulation from the pituitary gland. Estrogens and progesterone are key hormones that regulate healthy endometrium physiology and are essential for creating a microenvironment conducive to embryo implantation. Adenomyosis is thought to be caused by an imbalance in hormonal signaling between these two hormones (Rossi *et al.* 2022, Vannuccini & Petraglia 2022). Although hyperestrogenism is commonly cited as a significant factor in the development of adenomyosis, few studies have investigated the role of progesterone resistance in adenomyosis-related infertility (Vannuccini & Petraglia 2022, d’Argent *et al.* 2023). One proposed molecular cause of progesterone resistance is a reduced expression of progesterone receptors, found in endometriotic lesions and eutopic endometrium from women with endometriosis (Nie *et al.* 2009, Shen *et al.* 2015, Wölfler *et al.* 2016). This endometrial progesterone unresponsiveness may negatively influence both the proliferation of endometrial cells and the decidualization of stromal cells, leading to both lesion growth and a non-receptive endometrium (Maclean & Hayashi 2022, Zhang & Wang 2023). In our mice affected by adenomyosis, the PGR expression was reduced in the stromal and glandular cells of the uterus of 3-month-old mice affected by adenomyosis. This finding suggests that it could be a potential mechanism leading to adenomyosis-related infertility, which was previously observed in the study of Mehasseb *et al.* (Mehasseb *et al.* 2011). Additionally, their research revealed that women with adenomyosis exhibit an inadequate response to progesterone during the secretory phase, leading to an estrogen-driven hyperproliferation of endometrium due to an insufficient response to progesterone. By analyzing the expression of PGR in uterine stromal cells from 1-month-old mice (Supplementary Data, Fig. 1, see section on supplementary materials given at the end of this article), we indirectly investigate whether progesterone resistance plays a role in adenomyosis at an earlier stage of the disease. At 1 month of age, the mice respond appropriately to progesterone signaling, suggesting that progesterone unresponsiveness appears later in disease progression. Impaired progesterone responsiveness could contribute to endometrial dysfunction and the subsequent reduction in endometrial receptivity, vital for implantation and successful pregnancy. This phenomenon warrants further investigation to understand its mechanistic underpinnings and clinical implications. Further studies are needed to confirm progesterone resistance in this mice model, notably through evaluating the response of endometrial cells to progesterone stimulation.

Adenomyosis can also impair endometrial receptivity, a disorder associated with a lack of adequate expression of adhesion molecules, reduced expression of implantation markers, and altered function of genes involved in embryonic development (Vannuccini *et al.* 2017). Our analysis of implantation-related markers during the estrous phase revealed decreased *HoxA10* and *Itgb3* mRNA levels in adenomyotic uteri, particularly in 1-month-old mice. The reduction of *HoxA10* expression continued over time. Interestingly, the homeobox gene, known to be involved in the development of the female genital tract during the embryonic period and in regulating endometrial receptivity in adulthood, is a target of progesterone (Du & Taylor 2016). Decreased *HoxA10* endometrial expression correlates with lower endometrial receptivity, leading to reduced implantation rates (Zanatta *et al.* 2010). In previously published studies with adenomyosis-induced mice, *HoxA10* expression was assessed and shown to decrease during the implantation window when the endometrium is receptive to the embryo (Guo *et al.* 2018, Guan *et al.* 2022). Our present study provides evidence that *HoxA10* is decreased outside the implantation window, and the expression levels could be insufficient to ensure effective implantation and therefore successful pregnancy.

Epigenetic changes are thought to be responsible for reduced expression of progesterone receptor and homeobox genes in endometriosis (Wu *et al.* 2005, 2006). Further studies are needed to determine whether these epigenetic changes are also accountable for progesterone resistance and reduced *HoxA10* expression in adenomyosis. The transcription factor *HoxA10*, in response to progesterone, can enhance integrin *β*3 expression during the luteal phase of menstrual cycle (Joshi *et al.* 2021). Integrin β3 is a cell adhesion molecule indispensable for successful implantation during uterine receptivity and therefore overexpressed in the endometrium during the receptive window (Lessey *et al.* 1994). Just as with the analysis of *HoxA10*, we similarly observed a decrease in the gene expression of integrin β3 in the uteri of 1-month-old mice with adenomyosis when compared to the control group during the estrous phase, mirroring what was observed during the implantation window in mice induced for adenomyosis (Guo *et al.* 2018, Guan *et al.* 2022). For patients undergoing ART, analysis of endometrial integrin β3 expression is critical, as it has been shown that ART is less effective if the endometrial level of integrin β3 is low (Joshi *et al.* 2021). Another essential key factor for embryo implantation is *Lif*. This cytokine has multiple roles: it influences endometrial receptivity, affects trophoblastic function, and participates in placental vascular formation (Xu *et al.* 2012). Our data indicate a significant increase in *Lif* expression in the uterus of adenomyosis-induced mice during the estrus phase of the estrous cycle. This result contrasts those found in previous works reporting lower *Lif* expression in the endometrium of mice or women with adenomyosis during the implantation window, potentially explaining the low implantation rate in these experimental groups (Yen *et al.* 2017*b*, Guo *et al.* 2018). In our results, the increase in *Lif* mRNA expression could suggest a compensatory response that may reflect the uterus’ attempt to counteract the diminished implantation markers such as *HoxA10* and integrin β3. A study on adenomyosis-derived organoids showed the same trend as that in our adenomyosis mice, i.e. increased *Lif* expression (Juárez-Barber *et al.* 2022). The authors hypothesized that *Lif* could play a role not only in the implantation process but also in epithelial–mesenchymal transition through the regulation of the Wnt/b-catenin pathway (Rosario & Stewart 2016, Juárez-Barber *et al.* 2022). These findings are crucial for understanding the various mechanisms involved in the physiopathology of adenomyosis and associated symptoms such as infertility and thus finding suitable therapeutic regimens. In our study, the expression of implantation-related markers was evaluated outside the implantation window, revealing dysregulation of these genes before any potential pregnancy.

This study's major challenge was identifying whether all the described effects are the consequence of adenomyosis or caused by neonatal tamoxifen treatment. Neonatal treatment with tamoxifen (TAM) has been used extensively for examining adenomyosis pathogenesis and drug screening (Parrott *et al.* 2001, Yen *et al.* 2017*a*, Marquardt *et al.* 2020). While it is true that TAM could impair adult rodent ovarian function, results from the literature are controversial. TAM administration in adult mice did not affect the number of primordial and primary follicles but decreased the number of growing follicles (Akduman *et al.* 2014). TAM was also shown to activate dormant primordial follicles transiently (Wei *et al.* 2022). No alteration of the follicle number or prevalence of granulosa apoptosis was found in rats (Nynca *et al.* 2023). These results do not reflect the neonatal TAM treatment to induce adenomyosis in mice. To our knowledge, no study has demonstrated the negative effect of neonatal tamoxifen administration on ovarian function. Interestingly, TAM-treated breast cancer survivors did not have decreased ovarian reserve compared to TAM non-users (Shandley *et al.* 2017). Therefore, the direct or indirect impacts of observed alterations on fertility reduction remain unanswered. Nevertheless, adenomyosis has been reported in 60% of postmenopausal women taking tamoxifen therapy for an extended period, suggesting the promotional effect of tamoxifen in establishing adenomyosis lesions (Harada *et al.* 2016). We are confident in the fact most features found in women suffering from adenomyosis, such as the decrease in menstrual cycle length, the earlier age of the menarche, and the effect on ovarian reserve are reflected in the murine model (Templeman *et al.* 2008, Guo *et al.* 2018).

In conclusion, our study provides insights into adenomyosis’ impact on female reproductive health. The disrupted estrous cyclicity, the compromised ovarian follicle development, the reduced fertility and progeny, the decreased expression of endometrial progesterone receptor and receptivity-related genes collectively suggest a potential contribution to the subfertility observed in adenomyosis. These findings underscore the need for further research into the mechanisms underlying these effects and the potential development of targeted interventions to improve fertility outcomes in women affected by adenomyosis.

## Supplementary Materials

Supplementary data. Figure 1. Progesterone receptor (PGR) expression in uteri of 1 month-old control and adenomyosis-induced mice.

## Declaration of interest

The funders had no role in the design of the study; in the collection, analyses, or interpretation of data; in the writing of the manuscript; or in the decision to publish the results.

## Funding

This research was funded by the Fonds de la Recherche Scientifique – (F.R.S.-FNRS, Belgium)http://dx.doi.org/10.13039/501100002661, grant number T.0171.21 and J.0143.22, the Foundation Léon Fredericq (University of Liège), grant number FSR-F-2022-FM.

## Author contribution statement

Conceptualization, C.M. and M.S.; methodology, C.M. and M.S.; validation, M.S., J.V and C.M.; formal analysis, investigation, M.S., J.V., S.B., J.B., and L.B.; resources, M.S., J.V., J.B., L.B., and C.M., writing – original draft preparation, M.S. and C.M.; writing – review and editing, M.S., C.M., J.B., L.B., M.N., and J.V.; supervision, C.M., project administration, M.S., J.V., and C.M.; funding acquisition, C.M. and M.N. All authors read and agreed to the final version of the manuscript.
