# Supplementary material for: Impaired fertility in adenomyosis: a murine model reveals endometrial receptivity and progesterone resistance imbalances
Source: Reproduction. 2024 Apr 17;167(5):e240019. doi: 10.1530/REP-24-0019 (PMC11056956; doi:10.1530/REP-24-0019)
Supplement: Supplementary data. Figure 1. Progesterone receptor (PGR) expression in uteri of 1 month-old control and adenomyosis-induced mice. [file supplementary_figure_1.pdf]

SUPPLEMENTARY DATA

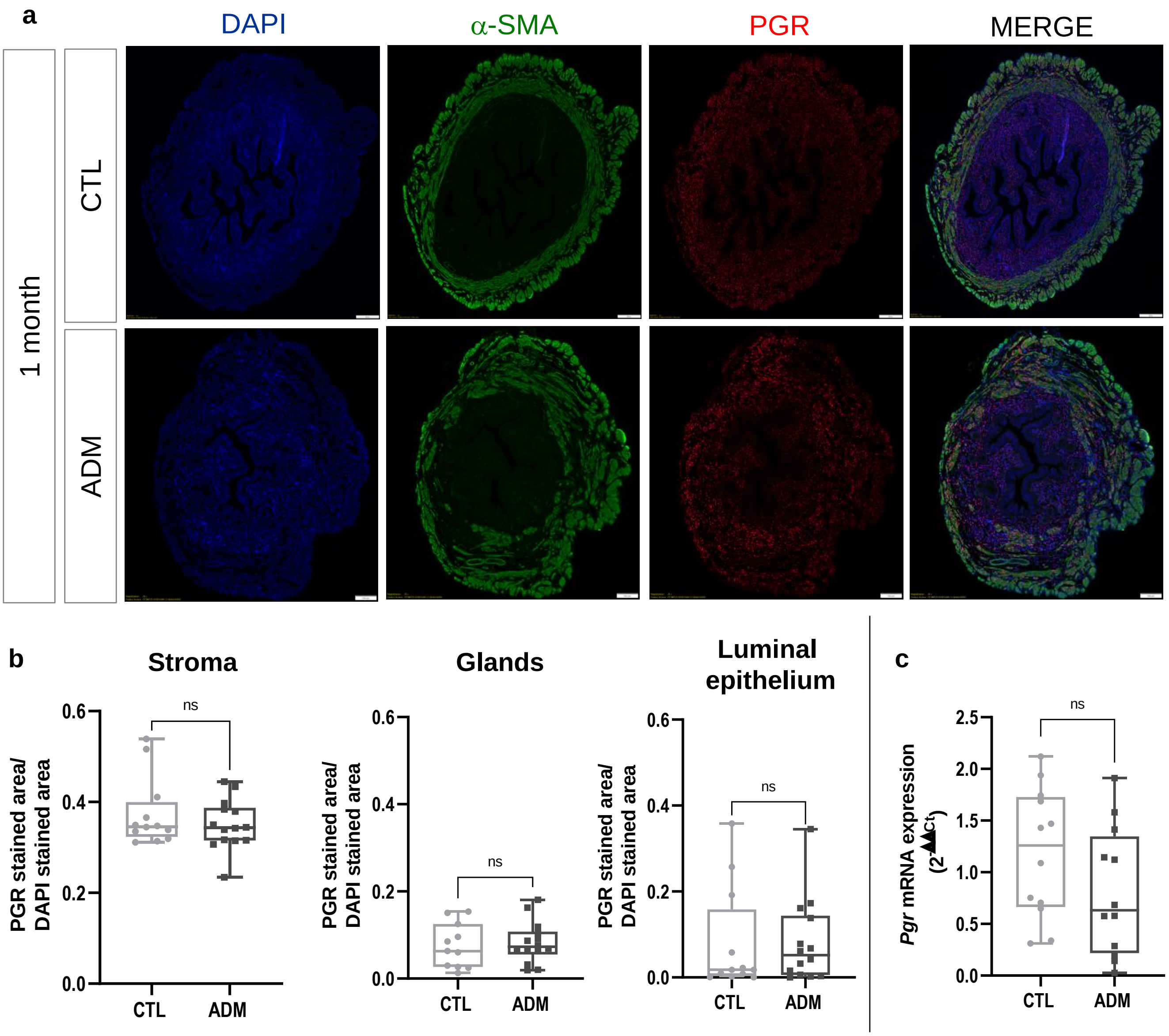

Supplementary data. Figure 1. Progesterone receptor (PGR) expression in uteri of 1 month-old control and adenomyosis-induced mice.

Immunofluorescence **(a)** and computer-assisted quantification **(b)** of PGR expression in uteri of 1-month-old control and adenomyosis-induced mice. Bar scale = 200µm (CTL), Bar scale = 100µm (ADM). The mRNA expression levels of *Pgr* were also assessed **(c)**. The values are the median with the interquartile range. Statistical significance (Mann-Whitney test): ns = non-significant
